# Contemporary carotid artery stenting practices and peri-procedural outcomes in different European countries: ROADSAVER study multicentric insights

**DOI:** 10.1186/s42155-025-00528-z

**Published:** 2025-04-12

**Authors:** Stefan Müller-Hülsbeck, Zsolt Vajda, Piotr Odrowąż-Pieniążek, Zoltán Ruzsa, Roel Beelen, Aleksandar Gjoreski, Koen Deloose, Sérgio Castro, Benjamin Faurie, Alejandro Tomasello Weitz, Arne Schwindt, Paweł Latacz, Antonio Orgaz Pérez-Grueso, Vladimir Cvetić, Ralf Langhoff, Sasko Kedev, all ROADSAVER study investigators

**Affiliations:** 1 Department of Diagnostic and Interventional Radiology and Neuroradiology, DIAKO Hospital GmbH, Academic Teaching Hospital Christian-Albrechts-University Kiel – Faculty of Medicine, Deaconess Hospital Flensburg, Knuthstraße 1, 24939 Flensburg, Germany; 2 Neurovascular Unit, Moritz Kaposi Teaching Hospital, Kaposvár, Hungary; 3 Department of Radiology, Fejér County Szent György University Teaching Hospital, Székesfehérvár, Hungary; 4 Department of Interventional Cardiology, Institute of Cardiology, Jagiellonian University, Medical College, Krakow, Poland; 5 Department of Vascular Surgery Division on Endovascular Therapy, John Paul II Hospital, Krakow, Poland; 6 Bács-Kiskun County Hospital, Teaching Hospital of the Szent-Györgyi Albert Medical University, Kecskemét, Hungary; 7 Department of Internal Medicine, Division of Invasive Cardiology, University of Szeged, Szeged, Hungary; 8 Department of Vascular and Thoracic Surgery, O.L.V. Aalst, Aalst, Belgium; 9 Department for Diagnostic and Interventional Radiology, Clinical Hospital “Acibadem Sistina”, Skopje, North Macedonia; 10 Department of Vascular Surgery, AZ-Sint Blasius, Dendermonde, Belgium; 11 Department of Imagiology, Interventional Neuroradiology Unit, Centro Hospitalar Vila Nova de Gaia/Espinho, Vila Nova de Gaia, Portugal; 12 Infirmerie Protestante de Lyon, Caluire-Et-Cuire, France; 13 Interventional Neuroradiology Section, Department of Radiology, Vall d’Hebron University Hospital, Barcelona, Spain; 14 Department of Vascular Surgery, St. Franziskus-Hospital, Münster, Germany; 15 Department of Vascular Surgery and Angiology, Brothers of Mercy St. John of God Hospital, Kraków, Poland; 16 Servicio de Angiología y Cirugía Vascular, Hospital Universitario de Toledo, Toledo, Spain; 17 Cardiovascular Radiology Department, Clinic for Vascular and Endovascular Surgery, University Clinical Centre of Serbia, Belgrade, Serbia; 18 Department of Angiology, Brandenburg Medical School Theodor Fontane, Campus Clinic Brandenburg, Brandenburg an der Havel & Sankt Gertrauden – Hospital, Berlin, Germany; 19 Department of Cardiology, Faculty of Medicine, University Clinic of Cardiology, University of St. Cyril & Methodius, Skopje, North Macedonia

**Keywords:** Carotid artery, Carotid stenosis, Angioplasty, Stents, Stroke prevention, Geographical variation

## Abstract

**Background:**

Regional variations in patient selection and procedural techniques for carotid artery stenting have been well documented. However, their impact on procedural outcomes, especially with the use of dual-layer micromesh stents, is not fully understood.

**Methods:**

This prospective, multi-center observational study included 1965 patients with asymptomatic or symptomatic carotid artery stenosis treated with the Roadsaver dual-layer micromesh stent. The primary outcome measure was the 30-day rate of major adverse events, defined as any death or stroke occurring within 30 days post-procedure. This sub-analysis compared patient characteristics and procedural techniques across 13 participating countries and investigated differences in outcomes via logistic regression modelling.

**Results:**

Patient demographics, comorbidities, and symptom presentation varied widely among countries. Similarly, the frequency of use of duplex ultrasound and diffusion-weighted magnetic resonance imaging at baseline and 30-day follow-up differed. Procedural approaches also varied, with differences in femoral access site selection (18.2% to 100.0%), use of embolic protection devices (0.0% to 100.0%), pre-dilatation (4.3% to 46.7%) and post-dilatation (66.7% to 100.0%). Although 30-day major adverse event rates differed across the compared countries, after adjusting for post-dilatation balloon pressure (categorized as no post-dilatation vs. ≤ 11atm vs. > 11atm), and the number of enrolled patients per study site, the difference became statistically non-significant.

**Conclusion:**

Our study reveals variability in patient selection, procedural carotid stenting practices and clinical outcomes across European countries. The differences in 30-day any death or stroke rates between countries may be attributed to differing post-dilatation practices and the number of enrolled patients per study site.

**Level of evidence:**

Level 3, observational study.

**Trial registration:**

Clinicaltrials.gov identifier: NCT03504228.

**Visual Abstract:**

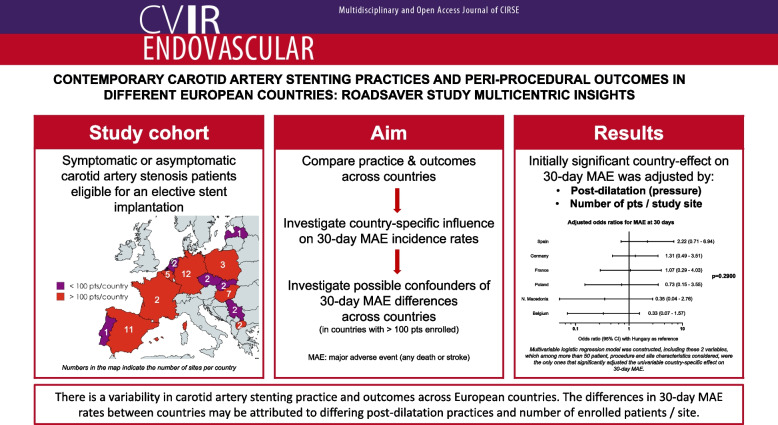

**Supplementary Information:**

The online version contains supplementary material available at 10.1186/s42155-025-00528-z.

## Introduction

Carotid endarterectomy (CEA) and carotid artery stenting (CAS) treat carotid artery stenosis to reduce ischemic stroke risk. CAS is less invasive than CEA, with lower risks of myocardial infarction and cranial nerve palsy, but higher distal embolization risk, causing slightly more peri-procedural minor, non-disabling strokes [1–3]. Advances in patient selection, techniques, carotid stent design and adjunctive devices have improved CAS outcomes [4]. New-generation dual-layer micromesh carotid artery stent(s) (DLMS), like Roadsaver™ Carotid Stent system, provide sustained cerebral embolic protection and are more effective in reducing cerebral microembolisms compared to earlier single-layer stents, like Carotid Wallstent [5].

Substantial geographical variation in the utilization of CAS and CEA has been observed in the past [6]. An analysis of the Vascular Quality Initiative (VQI) database, which included 57,555 carotid artery revascularization procedures from 2009 to 2015, revealed significant regional variations in patient selection and operative techniques, encompassing not only the choice between CEA and CAS, but also patient symptomaticity, imaging modality for procedure planning, and pharmacotherapy. In CAS context, variability was also noted in embolic protection device (EPD) use [7].

Data on regional variation in practice and outcomes associated with contemporary CAS is limited. Therefore, we evaluated the country-specific differences in patient selection, procedural techniques and outcomes in a large European cohort undergoing elective treatment with a DLMS as per local routine practice. The cohort's 30-day outcomes, well below the strictest guideline thresholds, have recently been reported for both asymptomatic and symptomatic patients [8]. To complement these data, this analysis aimed to investigate country-specific influence on 30-day MAE incidence rate and identify confounders of possible differences.

## Methods

### Study design & population

The ROADSAVER is a prospective, multi-center, observational study. For more details on the study design, see [9]. Patient enrollment occurred from January 2018 to February 2021. In total, 1965 asymptomatic or symptomatic patients with a non-occlusive, non-thrombotic carotid artery stenosis eligible for elective CAS were enrolled and treated with the study device. For a full list of participating sites and investigators, see the Supplementary Materials. Enrollment was contingent upon written informed consent and successful guidewire passage through the target lesion. Each participating site received Ethics Committee approval for the study in accordance with local regulations, as applicable. Terumo Europe sponsored the study.

### Procedure details

Patient work-up and procedures followed each hospital’s standard practices, including imaging, pre-/post-dilatation, use of EPDs, and pharmacological management (e.g. for vasospasm, hypotension, arrhythmias and intra- and post-procedural antithrombotic medication). NASCET criteria determined angiographic stenosis severity before and after the procedure [10].

### Study device

All patients were treated with the Roadsaver™ Carotid Stent System (MicroVention Europe, subsidiary of Terumo Corporation), a self-expanding, nitinol DLMS. The 5-Fr rapid exchange delivery catheter features a low crossing profile (1.7 mm diameter). The stent has a dual-layer braided design with an outer layer comprised of a flexible and conformable closed cell structure, while the inner layer forms a micromesh with 375–700 micron-sized pores. The stent is re-sheathable up to 50% deployment allowing repositioning.

### Outcomes

The primary endpoint was the 30-day incidence of major adverse events (MAE), defined as the combined occurrence of any death or stroke within 30 days post-procedure. A stroke was characterized as an acute neurological condition with focal symptoms and signs persisting for 24 h or more. An independent clinical events committee adjudicated all reported deaths, strokes, and carotid artery revascularizations.

### Enrolling countries

The study included 52 sites from 13 European countries with the number of patients and the number of study sites indicated between brackets: Hungary (501/7), Germany (347/12), Belgium (234/5), Poland (186/3), Spain (180/11), France (146/2), North Macedonia (140/2), Slovakia (83/2), Portugal (69/1), Czech Republic (45/2), Latvia (20/1), Serbia (8/2) and the Netherlands (6/2).

### Statistical analysis

Descriptive statistics included means with standard deviations for continuous data and percentages for categorical data. A univariable logistic regression model investigated country-specific influence on 30-day MAE incidence rates, restricting the analysis to countries with at least 100 enrolled subjects. The model was then individually adjusted for approximately 50 patient or procedural characteristics (listed in the Supplementary Materials) to explore whether differences in these characteristics across countries could explain any univariable country-specific effect on MAE rates. Results were presented as odds ratios with 95% confidence intervals by country, using Hungary as the reference country. Hungary was chosen for its middle-ranked MAE rate among analyzed countries. Characteristics were considered to significantly adjust the country-specific effect if the latter became non-significant at the 5% level. Categorical variables with a "Yes/No/Unknown" response option and low "Unknown" counts were collapsed into the "No" category to improve model fit. A final multivariable model was constructed including only those variables that significantly adjusted the univariable country-specific effect. Note that this model is not a fully adjusted predictive model for 30-day MAE rates but is designed to investigate country-specific effects and potential inter-country differences driving these effects. Statistical analyses were performed using SAS software, version 9.4 (SAS Institute Inc., Cary, NC, USA).

## Results

There was a substantial heterogeneity in patient demographic and procedural characteristics across countries. The proportion of subjects aged ≥ 75 years varied from 0% (Serbia) to 48.6% (France), while female representation ranged from 12.5% (Serbia) to 35.0% (Latvia). Symptomatic subjects comprised between 13.7% (France) and 95.7% (Portugal) of the sample. Diabetes mellitus prevalence ranged from 10.0% (Latvia) to 40.0% (Czech Republic). Previous stroke rates were the lowest in Latvia (10.0%) and the highest in Portugal (55.1%) (Table 1).

**Table 1:** Selected patient characteristics per country

Country	N	Symptomatic subjects	Age ≥ 75 years	Sex (Female)	DM	AH	Current smoker	Hyperlipidemia	Previous stroke	PVD	CVD
All countries	1965	49.4	35.9	29.6	32.1	87.4	25.2	76.0	33.4	26.8	38.3
**Hungary**	501	52.1	28.5	34.5	34.1	90.6	28.3	72.7	43.9	20.2	28.7
**Germany**	347	33.4	44.7	28.2	35.1	88.8	29.3	81.8	19.9	28.8	47.8
**Belgium**	234	47.9	47.4	29.5	28.8	80.8	19.3	81.2	23.9	34.2	41.9
**Poland**	186	36.0	31.7	30.6	30.1	**96.2**	22.0	**96.8**	40.9	24.2	57.0
**Spain**	180	88.9	37.2	19.4	36.3	**71.1**	20.6	63.3	53.9	11.7	**15.0**
**France**	146	**13.7**	**48.6**	28.8	30.8	80.1	20.5	74.7	11.6	31.5	50.0
**North Macedonia**	140	72.9	22.9	32.9	30.0	95.7	27.1	80.7	30.0	**66.4**	37.9
Slovakia	83	43.4	21.7	27.7	25.3	89.2	21.7	**38.6**	12.0	21.7	39.8
Portugal	69	**95.7**	42.0	27.5	34.8	88.4	26.1	62.3	**55.1**	**7.2**	17.4
Czech Republic	45	33.3	28.9	24.4	**40.0**	95.6	44.4	82.2	51.1	15.6	51.1
Latvia	20	30.0	30.0	**35.0**	**10.0**	95.0	5.0	75.0	**10.0**	30.0	30.0
Serbia	8	75.0	**0.0**	**12.5**	14.3	87.5	**50.0**	87.5	37.5	50.0	**87.5**
Netherlands	6	66.7	16.7	16.7	16.7	83.3	**0.0**	83.3	50.0	16.7	66.7

Table shows frequencies (%) per country. N: number of patients per country. Countries are listed as per the number of enrolled patients in a descending order. Highlighted in bold are countries with >100 patients enrolled or minimum/maximum values for each characteristic/variable

*AH* Arterial hypertension, *CVD* Cardiovascular disease, *DM* Diabetes mellitus, *PVD* Peripheral vascular disease

Duplex ultrasound (DUS) is a diagnostic imaging tool for carotid artery stenosis assessment, used in patient work-up before CAS and later follow up. In this study, DUS was performed in over 80% of the patients at baseline and/or at 30-days in most countries. Interestingly, while North Macedonia had 100% DUS evaluations at both time points, Serbia, Slovakia and Czech Republic displayed relatively low baseline DUS usage. Diffusion-weighted magnetic resonance imaging (DW-MRI), used to identify clinically silent ischemic cerebral microembolisms, was infrequently utilized. Most countries performed DW-MRI in less than 50% of patients at baseline and/or at 30-days, except for Portugal, Germany, and Serbia, where its use at baseline was slightly more frequent (Table 2). The data on other commonly used imaging modalities in the context of CAS were not collected in this study.

**Table 2:** Use of imaging modalities at baseline and 30-day follow up per country

Country	N	Baseline DUS	30-day DUS	Baseline DW-MRI	30-day DW-MRI
All countries	1965	75.5	87.6	30.0	11.2
**Hungary**	501	58.7	86.8	32.5	15.3
**Germany**	347	85.6	83.6	50.1	2.1
**Belgium**	234	78.6	93.4	20.1	1.3
**Poland**	186	94.6	98.9	**0.0**	**0.0**
**Spain**	180	81.7	62.1	32.8	7.5
**France**	146	93.2	95.8	38.4	37.8
**North Macedonia**	140	**100.0**	**100.0**	22.9	20.4
Slovakia	83	27.7	98.7	10.8	**0.0**
Portugal	69	78.3	82.4	56.5	45.6
Czech Republic	45	26.7	84.4	**0.0**	**0.0**
Latvia	20	75.0	100.0	20.0	**0.0**
Serbia	8	**12.5**	**0.0**	**87.5**	**50.0**
Netherlands	6	83.3	66.7	**0.0**	**0.0**

Table shows frequencies (%) per country. N: number of patients per country. Countries are listed as per the number of enrolled patients in a descending order. Highlighted in bold are countries with >100 patients enrolled or minimum/maximum values for each imaging modality at each time-point of evaluation

*DUS* Duplex ultrasound, *DW-MRI* Diffusion-weighted magnetic resonance imaging

Femoral access was commonly used and applied in 10 out of 13 countries in over 80% of the patients. In contrast, in Hungary radial access was most common (78.8%). Uniquely, the cervical approach was used in 20.6% of the patients in Spain. Use of EPDs was common and 7 countries used EPDs in 80% or more of the procedures. Very low rates of EPD use were noted in Hungary (29.5%) and The Netherlands (0). Pre-dilatation utilization was in general low with only 5 out of 13 countries using it in more than 30% of cases, with rates varying from 4.3% (Portugal) to 46.7% (Czech Republic). In contrast, post-dilatation use was highly prevalent, with 10 out of 13 countries applying it in over 90% of procedures. Its slightly lower use was noted in Spain (86.7%), Latvia (80%) and The Netherlands (66.7%). During pre-dilatation and post-dilatation, 3 mm and 5 mm ballons, respectively were most often used. Highest balloon pressures for both pre- and post-dilatation were used by Polish and lowest by Czech, Serbian and centers in the Netherlands (Table 3 and Supplementary Materials; Figure S1)

**Table 3:** Selected procedural characteristics per country

Country	N	Femoral access	Radial access	Other access	Closure device	Embolic protection	Pre-dilatation	Post-dilatation
All countries	1965	70.3	26.3	3.5	63.9	63.8	25.6	96.1
**Hungary**	501	**18.2**	**78.8**	3.0	34.7	29.5	7.2	99.4
**Germany**	347	99.1	0.3	0.6	88.2	71.5	45.5	93.9
**Belgium**	234	96.2	2.1	1.7	91.0	56.8	15.8	97.9
**Poland**	186	92.5	7.5	**0.0**	34.4	**100.0**	44.6	**100.0**
**Spain**	180	65.6	13.9	**20.6**	61.7	85.6	46.1	86.7
**France**	146	82.2	11.6	6.2	78.8	99.3	15.1	96.6
**North Macedonia**	140	60.7	39.3	**0.0**	80.0	47.9	34.3	96.4
Slovakia	83	**100.0**	**0.0**	**0.0**	77.1	48.2	10.8	98.8
Portugal	69	94.2	5.8	**0.0**	94.2	92.8	**4.3**	95.7
Czech Republic	45	97.8	**0.0**	2.2	15.6	97.8	**46.7**	93.3
Latvia	20	**100.0**	**0.0**	**0.0**	95.0	80.0	5.0	80.0
Serbia	8	**100.0**	**0.0**	**0.0**	**0.0**	**100.0**	25.0	**100.0**
Netherlands	6	**100.0**	**0.0**	**0.0**	**100.0**	**0.0**	16.7	**66.7**

Table shows frequencies (%) per country. N: number of patients per country. Countries are listed as per the number of enrolled patients in a descending order. Highlighted in bold are countries with >100 patients enrolled or minimum/maximum values per procedural variable. Category “radial access” may include ulnar access cases, while category “other access” may include brachial and/or cervical, depending on the country

**Fig. 1:**
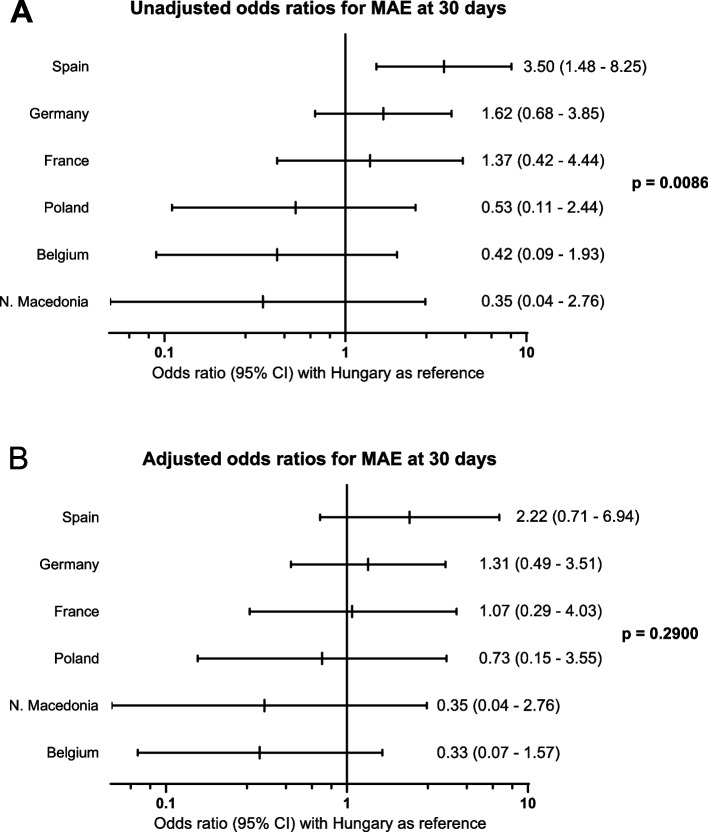
Geographical variation in 30-day major adverse event (MAE) incidence rates following carotid artery stenting (CAS) across European countries. Unadjusted (**A**) and adjusted (**B**) odds ratios (OR) with 95% confidence intervals (95% CI) for 30-day MAE following CAS. Analysis includes countries with at least 100 enrolled subjects and Hungary serves as the reference country (OR = 1.0). Logistic regression models were used to investigate country-specific influence on MAE incidence rates. The adjusted model includes the number of enrolled patients by center and the post-dilatation balloon pressure (categorized as no post-dilatation, ≤ 11 atm and > 11 atm). *P*-value indicates overall significance of country-specific variation

The 30-day MAE rates following CAS varied across European countries. Among countries that enrolled more than 100 patients, Spain showed the highest odds of 30-day MAE and North Macedonia, Belgium and Poland the lowest (Fig. 1A). Next, more than 50 patient, procedure and site characteristics were assessed as possible confounders of the country-specific effect on 30-day MAE. In a final multivariable model, the two variables that significantly adjusted the univariable country-specific effect were included, namely the number of enrolled patients by site and the categorical post-dilatation balloon pressure (no post-dilatation / post-dilatation with ≤ 11 atm / post-dilatation with > 11 atm). Following this adjustment, the observed country-specific differences in 30-day MAE rates were no longer statistically significant (Fig. 1B and Supplementary Materials; Figures S2, S3 and S4).

## Discussion

This large-scale, real-world observational CAS study offers valuable insights into patient selection, procedural techniques, and clinical outcomes across Europe. The observed variations in CAS practices underscore the importance of considering these differences when interpreting outcomes across regions. Importantly, the country-effect on 30-day MAE observed in the study was significanlty confounded by the volume of patients entrolled/treated at each site and varying post-dilatation practices accross countries.

The proportion of symptomatic patients in this study ranged from 13.7% to 95.7%, indicating differing intervention thresholds. In the VQI database, this ranged from 49 to 71% [7]. While revascularizing > 70% carotid stenosis in symptomatic patients is well established, benefits for asymptomatic patients have been questioned [3]. It was believed that asymptomatic patients on modern medical therapy had an annual stroke risk of around 1%, with CAS or CEA periprocedural risks potentially exceeding long-term benefits. However, a study by Howard et al. showed a strong correlation between ipsilateral carotid stenosis degree and stroke risk [11]. These findings imply that asymptomatic patients with higher degree stenosis (80–99%) benefit from revascularization, while those with moderate stenosis (50–69%) may not [11]. These findings were reflected in the 2021 European Stroke Organisation (ESO) guidelines on CEA and CAS, published after the ROADSAVER study enrollment concluded [12]. Thus, patient selection for CAS during the study was based on older evidence.

Differences in physician and patient preferences might impact treatment decisions and drive practice variability. The operator's specialty may also contribute to disparities in patient selection and procedural preferences [13, 14]. Additionally, differences in comorbidity prevalence may be due to differential selection criteria and variations in diabetes mellitus burden [15] and cardiovascular disease risk [16] across European countries.

Variation in imaging practices before and after CAS is corroborated by existing literature [7]. In this study, DUS utilization was widespread. This imaging modality is essential for assessing carotid stenosis before and after stenting, providing detailed imaging and hemodynamic information to evaluate stenosis severity and stenting success. The use of DW-MRI was less uniform, reflecting differences in clinical practice and resource availability across countries. DW-MRI identifies acute ischemic changes and assesses stroke risk, providing critical information for both pre-procedural planning and post-stenting evaluation. Although data on computed-tomography angiography (CTA) use was not collected, it is noteworthy that both DUS and CTA are recommended by CIRSE Standards of Practice on Carotid Artery Stenting [17]. Nevertheless, caution is warranted as discrepancies in stenosis severity assessment may occur, potentially leading to carotid stenosis misclassification and differences in qualification processes between centers [18].

Significant variations in procedural techniques were observed across countries, including the use of specific access sites, utilization of EPDs and pre-/post-dilatation practices. Despite increasing interest in the radial approach to reduce vascular and bleeding complications, it was prominently used only in Hungary and North Macedonia. Spain recorded a relatively high use of the trans-cervical approach. EPD use ranged from 0.0% to 100%, reflecting differing perceptions of effectiveness or specific patient or procedural factors [19]. This variability is surprising given evidence supporting increased peri-procedural stroke risk in unprotected carotid stent placement [17, 20, 21], highlighting that the EPD use in CAS is still contested, especially with new-generation DLMS use.

Pre-dilatation was less common, but when performed 3 mm balloons were used, as indicated by recent CAS practice standards [17]. In contrast, post-dilatation was highly prevalent. The CIRSE Standards of Practice Committee recommends post-dilatation only if the final angiogram after CAS shows residual stenosis exceeding 30% [17]. This recommendation is supported by studies suggesting an increased risk of neurological occurrences with post-dilatation [22–24]. However, these findings may mainly apply to first-generation single-layer stents, where the "cheese-grater effect", i.e., extrusion or dislodgement of plaque material through stent struts during implantation and post-dilatation, was suggested [25]. Double-layer micromesh stents, however, reduce the risk of large plaque prolapse after post-dilatation, even in unstable plaques [26].

A difference in outcomes across countries was observed, with Spain showing the highest odds of 30-day MAE among countries with more than 100 patients enrolled. Our modelling analyses suggest this may be due to low patient enrollment per study site in Spain and lower utilization of post-dilatation, often with lower balloon pressures (≤ 11 atm). Patient enrollment per site may indicate overall experience in carotid artery revascularization, a known factor influencing CAS outcomes [27]. These findings also highlight the importance of adequate post-dilatation, suggesting that with the Roadsaver DLMS a more liberal post-dilatation practice might be warranted to optimize CAS outcomes. Further research, however, is needed to identify the optimal post-dilatation approach in CAS, tailored to each patient and stent type. Importantly, this analysis aimed to investigate factors that may explain the observed country-effect, not to create a predictive model. Other patient and procedural factors may influence 30-day MAE rates as shown in the primary manuscript of the ROADSAVER study [8].

The observed heterogeneity in CAS practices underscores the need for standardization across Europe. Harmonizing patient selection criteria, imaging protocols, and procedural techniques could further improve clinical outcomes. The CIRSE Standards of Practice on Carotid Artery Stenting provide recommendations for patient selection, procedural techniques, and post-treatment care [17]. Similarly, a Clinical Consensus Statement of the European Society of Cardiology (ESC) Council on Stroke and the ESC Working Group on Aorta and Peripheral Vascular Diseases addresses stroke risk management in carotid atherosclerotic disease [25]. These documents could serve as a basis for further development of evidence-based guidelines.

### Limitations

Data on the frequency and outcomes of CEA, CAS and utilization of other stents than DLMS were not collected, preventing direct comparison of different techniques. Also, we did not collect data on computed tomography perfusion, other CTA techniques, nor different than DW-MRI sequences. The number of enrolled patients in some countries and study sites was low; to addresses this concern, only countries with > 100 patients were included in the MAE analysis. However, even with this approach the wide confidence intervals suggest considerable uncertainty in the estimates, possibly due to sample size limitations and/or outcome variability. The reported analyses did not focus on the detailed intra-country variability, the findings solely represent generalized synthesis per country level. Finally, information on reimbursement policies, which may influence treatment selection in certain healthcare systems and countries, was not collected.

## Conclusions

Our study reveals significant variability in patient selection criteria, CAS procedural techniques, and treatment outcomes across European countries. Differences in 30-day MAE rates are primarily attributed to the volume of CAS patients per center and the practice of post-dilatation, suggesting that center experience and more robust post-dilatation may improve outcomes of CAS with Roadsaver DLMS. Additionally, these findings imply that certain CAS practices should be tailored to the chosen stent implant.

## Supplementary Information

Supplementary Material 1: Figure S1-S4.

## Data Availability

All relevant data generated or analysed during this study are included in this published article and its supplementary information files.

## Abbreviations

CAS: Carotid artery stenting
CEA: Carotid endarterectomy
DUS: Duplex ultrasound
DLMS: Dual-layer micromesh stent
DW-MRI: Diffusion-weighted magnetic resonance imaging
EPD: Embolic protection device
MAE: Major adverse events
