# Supplementary material for: Contemporary carotid artery stenting practices and peri-procedural outcomes in different European countries: ROADSAVER study multicentric insights
Source: CVIR Endovasc. 2025 Apr 12;8:29. doi: 10.1186/s42155-025-00528-z (PMC11993519; doi:10.1186/s42155-025-00528-z)
Supplement: Supplementary file 1 — Supplementary Material 1: Figure S1-S4. [file 42155_2025_528_MOESM1_ESM.docx]

**Supplement to**

**Contemporary carotid artery stenting practices and peri-procedural outcomes in different European countries: ROADSAVER study multicentric insights**

[Participating sites and (co)investigators 1](#_Toc189178242)

[Statistical methods 6](#_Toc189178243)

[Figure S1: Pre- and post-dilatation balloon diameter size and pressure 9](#_Toc189178244)

[Figure S2: Number of patients per site by country 10](#_Toc189178245)

[Figure S3: Post-dilatation balloon pressure (categorized) 11](#_Toc189178246)

[Figure S4: Odds ratio for MAE adjusted for number of patients per site (A) and adjusted for the post-dilatation balloon pressure (B) 12](#_Toc189178247)

# Participating sites and (co)investigators

| **Country** | **Site** | **Investigator(s)** |
| --- | --- | --- |
| Belgium | Department of Vascular and Thoracic Surgery  Imelda Hospital Bonheiden  Bonheiden | Jürgen Verbist (PI)  Wouter Van Den Eynde |
| Belgium | Department of Medical Imaging  AZ Groeninge  Kortrijk | Olivier François (PI)  Tommy Andersson |
| Belgium | Department of Vascular Surgery  University Hospitals Leuven  Leuven | Kim Daenens (PI)  Sabrina Houthoofd |
| Belgium | Department of Vascular and Thoracic Surgery  O.L.V. Aalst  Aalst | Roel Beelen (PI)  Maene Lieven  Moerman Leslie  Isabel Bouckenooghe |
| Belgium | Department of Vascular Surgery  AZ-Sint Blasius  Dendermonde | Koen Deloose (PI)  Joren Callaert |
| Czech Republic | Department of Radiology  University Hospital Ostrava  Ostrava   Faculty of Medicine  University of Ostrava  Ostrava | Tomáš Jonszta (PI)  Václav Procházka |
| Czech Republic | Department of Radiology  Military University Hospital Prague  Prague | Jiří Lacman (PI) |
| France | Centre de Cardiologie et d'Exploration de la Côte Basque  Bayonne | Jean-Luc Banos (PI)  Valerico Sanchez |
| France | Institut Cardiovasculaire  Grenoble | Benjamin Faurie (PI)  Serge Lanternier  Carmine Sessa  Dominique Chaix |
| Germany | Centre of Vasculare Medicine  Elblandklinikum  Radebeul | Torsten Fuß |
| Germany | SRH Zentralklinikum Suhl  Klinik für Innere Medizin I (Kardiologie  Angiologie und internistische Intensivmedizin)  Suhl | Volker Sesselmann (PI)  Torsten Vogel  Tina Zahlaus  Albert Woratcheck |
| Germany | Fuerst-Stirum Hospital  Cardiology and Vascular Medicine  Bruchsal | Martin Andrassy (PI)  Katrin Gegenheimer |
| Germany | Clinic for Vascular and Endovascular Surgery,   Pius-Hospital Oldenburg,   Carl von Ossietzky Universität,   Oldenburg | Jürgen Köhler (PI)  Christophe-Maria Ratusinski (Former PI)  Andreas Cöster |
| Germany | Department of Diagnostic and Interventional Radiology and Neuroradiology  DIAKO Hospital gGmbH  Flensburg | Stefan Müller-Hülsbeck (PI)  Michael Preiss  Leonardo Marques  Silke Hopf-Jensen |
| Germany | Department of Radiology and Neuroradiology  Klinikum Passau  Passau | Wiebke Kurre (PI) |
| Germany | Cardioangiologisches Centrum Bethanien at Agaplesion Bethanien Hospital  Frankfurt | Michael Piorkowski (PI)  Vladislav Ganchev |
| Germany | Ihre-Radiologen.de  Center for Diagnostic Radiology & Minimally Invasive Therapy  The Jewish Hospital  Berlin | Henrik Schröder (PI)  Ferdinand Rücker  Alexandre Lucas |
| Germany | Abteilung Gefäß- und Endovascular Chirurgie  Theresienkrankenhaus und Sankt Hedwig-Klinik Abteilung für Gefäßchirurgie  Mannheim | Matthias Tenholt (PI)  Maher Fattoum  Hamad Algedaiby  Domenico Marco Stillitano |
| Germany | Diagnostische Radiologie/Neuroradiologie  SRH Klinikum Karlsbad-Langensteinbach  Karlsbad | Christiane Pöckler-Schöniger (PI)  Fritz Bergen |
| Germany | Department of Vascular Surgery  St. Franziskus-Hospital  Münster | Arne Schwindt (PI)  Giovanni Torsello  Michel Bosiers |
| Germany | Department of Angiology  Brandenburg Medical School Theodor Fontane  Campus Clinic Brandenburg  Brandenburg an der Havel & Sankt Gertrauden – Hospital  Berlin | Ralf Langhoff (PI)  Andrea Behne  Mehmet Boral  David Hardung |
| Hungary | Központi Radiológiai Osztály  Markusovszky Egyetemi Oktatókórház Szombathely | Istvan Király (PI)  Csaba Nagy |
| Hungary | Neurosurgery Clinic  University of Szeged Hospital  Szeged | Pál Barzó (PI)  Tamas Nemeth   Markos-Gergely Gellerd |
| Hungary | Neurovascular Unit  Moritz Kaposi Teaching Hospital  Kaposvár   Department of Radiology  Fejér County Szent György University Teaching Hospital,  Székesfehérvár | Zsolt Vajda (PI)  Monika Szöts  Nagy Csaba Balazs  Zsuzsa Danyi |
| Hungary | Department of Interventional Radiology  Heart and Vascular Centre  Semmelweis University  Budapest | Balázs Nemes (PI) |
| Hungary | Bács-Kiskun County Hospital  Teaching Hospital of the Szent-Györgyi Albert Medical University  Kecskemét   Department of Internal Medicine  Division of Invasive Cardiology  University of Szeged  Szeged | Zoltán Ruzsa (PI)  Csavajda Ádám János |
| Hungary | Department of Neurosurgery  Neuroendovascular Division  University of Pécs  Pécs | Péter Csécsei (PI)  Alex Szolics (Former PI) |
| Latvia | Faculty of Medicine  Riga Stradiņš University  Riga   Department of Radiology  Riga Stradiņš University  Riga | Karlis Kupcs (PI)  Helmut Kidikas |
| Netherlands | Department of Radiology  Medical Imaging Center  University Medical Center Groningen  University of Groningen  Groningen | Reinoud Bokkers (PI)  Maarten Uyttenboogaart |
| Netherlands | Department of Radiology  St Antonius Hospital  Nieuwegein | Daniel van den Heuvel (PI)  Jan Albert Vos  Marc van Leersum |
| North Macedonia | Department for Diagnostic and Interventional Radiology  Clinical Hospital ”Acibadem Sistina”  Skopje | Aleksandar Gjoreski (PI)  Filip Risteski |
| North Macedonia | Department of Cardiology  Faculty of Medicine  University Clinic of Cardiology  University of St. Cyril & Methodius  Skopje | Sasko Kedev (PI)  Ivan Vasilev  Danica Petkoska |
| Poland | Department of Interventional Cardiology  Institute of Cardiology  Jagiellonian University  Medical College  Krakow  Department of Vascular Surgery Division on Endovascular Therapy  John Paul II Hospital  Krakow | Piotr Odrowąz-Pieniążek (PI)  Piotr Paluszek   Roman Machnik  Marcin Misztal |
| Poland | Vascular Surgery Clinical Department University Hospital   Krakow | Paweł Latacz (PI) |
| Poland | Clinic of Vascular and Internal Diseases  Dr. Jan Biziel University Hospital No. 2  Bydgoszcz | Karol Suppan (PI) |
| Portugal | Department of Imagiology  Interventional Neuroradiology Unit  Centro Hospitalar Vila Nova de Gaia/Espinho  Vila Nova de Gaia | Sérgio Castro (PI)  Manuel Ribeiro  Miguel Veloso  Pedro Barros  Marta Rodrigues  Sofia Figueiredo |
| Serbia | Cardiovascular Radiology Department   Clinic for Vascular and Endovascular Surgery  University Clinical Centre of Serbia  Belgrade | Momčilo Čolić (PI)  Vladimir Cvetić   Borivoje Lukić |
| Serbia | Center of Radiology  Clinical Centre of Vojvodina  Novi Sad | Viktor Till (PI)  Dragan Andjelic  Vedran Zigic |
| Slovakia | Department of Interventional Cardiology  Kardiocentrum Nitra s.r.o.  Nitra | Peter Blaško (PI)  Peter Kurray |
| Slovakia | Department of Interventional Radiology  CINRE s.r.o.  Bratislava | Ivan Vulev (PI)   Tibor Balázs |
| Spain | Interventional Neuroradiology Section  Department of Radiology  Vall d’Hebron University Hospital  Barcelona | Alejandro Tomasello Weitz (PI)  Marc Ribó  David Hernandez |
| Spain | Department of Interventional Neuroradiology  Hospital Clínico Universitario de Valladolid  Valladolid | Jorge Galván Fernández (PI)  Miguel Arturo Schuller Arteaga  Mario Martinez Galdámez  Mercedes de Lera Alfonso |
| Spain | Angiology and Vascular Surgery Department  Hospital Universitari Son Espases  Palma | Pascual Lozano Vilardell (PI)  Krystell Daniela Escoto |
| Spain | International Vascular and Endovascular Institute (IVEI)  Angiology and Vascular Surgery Department  Hospital Quirónsalud Campo de Gibraltar  Palmones (Cádiz) | Rubén Rodríguez Carvajal (PI)  Fernando Gallardo |
| Spain | Neuroradiology Department  Hospital Universitario A Coruña  A Coruña | José Luis Diaz Valiño (PI)  Enrique Buceta (Former PI) |
| Spain | Servicio de Angiología y Cirugía Vascular  Complejo Hospitalario de Jaén  Hospital Universitario Médico-Quirúrgico  Jaén | Francisco Javier Martínez Gámez (PI)  José Enrique Mata Campos  Elena Herrero Martinez |
| Spain | Stroke Unit. Department of Neurology  Hospital Dr Josep Trueta  Institut d'Investigació Biomèdica de Girona  Girona | Mikel Terceño Izaga (PI)  Saima Bashir  Laura Paul  Joaquín Serena  Yolanda Silva |
| Spain | Servicio de Angiología y Cirugía Vascular  Hospital Universitario de Toledo  Toledo | Antonio Orgaz Pérez-Grueso (PI)  Maria Pilar Lamarca Mendoza |
| Spain | Interventional Neuroradiology Section  Department of Radiology  Donostia University Hospital  Donostia-San Sebastian | José Angel Larrea Peña (PI)  Pedro Navia Alvarez  Javier Masso Romero  Alexandre Lüttich Uroz |
| Spain | Department of Interventional Neuroradiology  Hospital Clinic of Barcelona  Barcelona | Jordi Blasco (PI)  Juan Macho Fernandez (Former PI)  Antonio Lopez  Javier Moreno  Luis San Román |
| Spain | Department of Radiology  Section of Vascular and Interventional Radiology  Hospital Universitario de Canarias  La Laguna  Tenerife | Heliodoro Vallés González (PI)  Jorge Senkichi Uchiyamada |

# Statistical methods

Logistic regression modelling was used to investigate the country-specific effect on MAE incidence rates at 30 days. An initial unadjusted model suggested significant differences (at the 5% level) in 30-day MAE rates across the 7 countries included in the analysis. To investigate whether variations in patient, procedural or site characteristics across the countries could be contributing to these differences, the model was individually adjusted for each of the variables listed below. Of these variables, the number of patients enrolled per site and post-dilatation balloon pressure each individually adjusted the country-specific effect to become non-significant at the 5% level.

**Patient Characteristics tested:**

Age (categorized as <75 years vs. ≥75 years)

Presence of Diabetes Mellitus in medical history (categorized as Yes vs. No*)

Type of Diabetes Mellitus in medical history (categorized as Type I vs. Type II vs. None*)

Presence of Hypertension in medical history (categorized as Yes vs. No*)

Presence of Hyperlipidemia in medical history (categorized as Yes vs. No*)

Presence of any Intracranial Pathology in medical history (categorized as Yes vs. No*)

Presence of Cardiovascular Disease in medical history (categorized as Yes vs. No*)

Myocardial Infarction in medical history (categorized as Yes vs. No*)

Presence of Arrhythmia in medical history (categorized as Yes vs. No*)

Presence of Extracranial Supra-aortic Disease in medical history (categorized as Yes vs. No*)

Presence of Valvular Heart Disease in medical history (categorized as Yes vs. No*)

Family history of Atherosclerosis (categorized as Yes vs. No vs. Unknown)

Obesity (categorized as Yes vs. No*)

Previous Stroke (categorized as Yes vs. No vs. Unknown)

Transient Ischemic Attack in medical history (categorized as Yes vs. No vs. Unknown)

Presence of Amaurosis Fugax in medical history (categorized as Yes vs. No vs. Unknown)

Smoking history (categorized as Current vs. Former vs. Never)

Neurological Status at baseline (Symptomatic vs. Asymptomatic)

MRI findings at baseline (categorized as Yes vs. No. vs. N/A (no MRI))

NIHSS Score at baseline (categorized as 0 vs. 1-4 vs. >4)

Type of Aortic Arch (categorized as Type I/Type II vs. Type III/Bovine)

Calcified Aortic Arch (categorized as Yes vs. No*)

Diseased Aortic Arch (categorized as Yes vs. No vs. Unknown)

Reference vessel diameter (RVD) – proximal (per mm increase)

Reference vessel diameter (RVD) – distal (per mm increase)

Lesion Length (per 5mm increase)

Diameter stenosis (%) pre-procedure (per 5% increase)

Calcified lesion (categorized as Yes vs. No*)

Ulcerated lesion (categorized as Yes vs. No vs. Unknown)

Irregular surfaced lesion (categorized as Yes vs. No*)

Target vessel tortuosity >90 degrees (categorized as Yes vs. No*)

Eccentricity (categorized as Concentric vs. Eccentric vs, Unknown)

Any lesion complexity (categorized as Yes vs. No)

**Procedural Characteristics tested:**

Access Site (categorized as Femoral vs. Radial (including Ulnar))**

Lesion location (categorized as CCA vs. ICA/Bifurcation)

Embolic Protection Device use (categorized as Yes vs. No)

Type of Embolic Protection Device (categorized as Distal vs. Proximal vs. None)

Pre-dilatation (categorized as Yes vs. No*)

Pre-dilatation balloon size (categorized as ≤3mm vs. >3mm vs. N/A (i.e. no pre-dilatation))

Pre-dilatation balloon pressure categorized as ≤9atm vs. >9atm vs. N/A (i.e. no pre-dilatation))

Post-dilatation (categorized as Yes vs. No)

Post-dilatation balloon size (categorized as ≤4.5mm vs. >4.5mm-≤5.5mm vs. >5.5mm vs. N/A (i.e. no post-dilatation))

Post-dilatation balloon pressure (categorized as ≤11atm vs. >11atm vs. N/A (i.e. no post-dilatation))

Stent Length (per 5mm increase)

Number of Stents (categorized as 1 vs. >1)

Deployed at Target (categorized as Yes vs. No)

Re-sheathed (categorized as Yes vs. No)

DAPT use post-procedure (categorized as Yes vs. No)

Diameter stenosis (%) post-procedure (per 5% increase)

Diameter stenosis (%) post-procedure (categorized as <30% vs. ≥30%)

**Site Characteristics tested:**

Number of enrolled subjects at site

* Where “Unknown” counts were low (< 100) these were collapsed into the “No” (or “None”) category.

** Other access sites (including brachial (n=31) and cervical (n=37)) were excluded from the analysis as there were no MAE to 30 days using these access sites which resulted in poor model conversion. Note that all cervical access sites were reported from Spain.

MRI = Magnetic Resonance Imaging, NIHSS = National Institutes of Health Stroke Scale, N/A = Not applicable, mm = millimeter, atm = atmosphere.

# Figure S1: Pre- and post-dilatation balloon diameter size and pressure


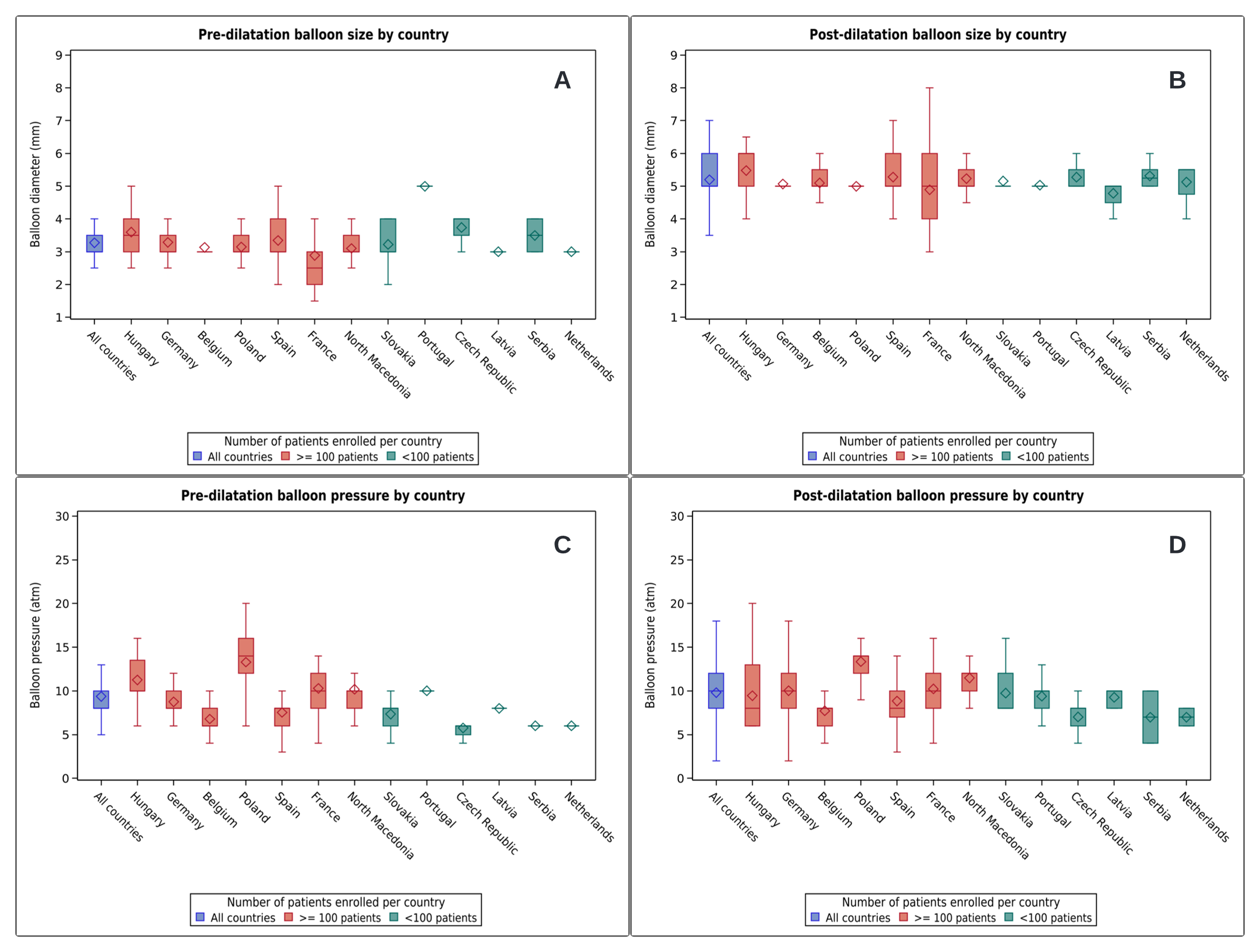


Figure shows the distribution of balloon diameters (A and B) and pressures (C and D) pre- and post-dilatation by country for patients in whom the lesion was pre- or post-dilated. The box indicates the interquartile range (IQR); the line inside the box indicates the median; and the diamond indicates the mean. The whiskers indicate the range of values outside the IQR but within 1.5x IQR. The boxes in blue are the data for all countries; red are the countries with > 100 patients; green are the countries with < 100 patients.

# Figure S2: Number of patients per site by country

**
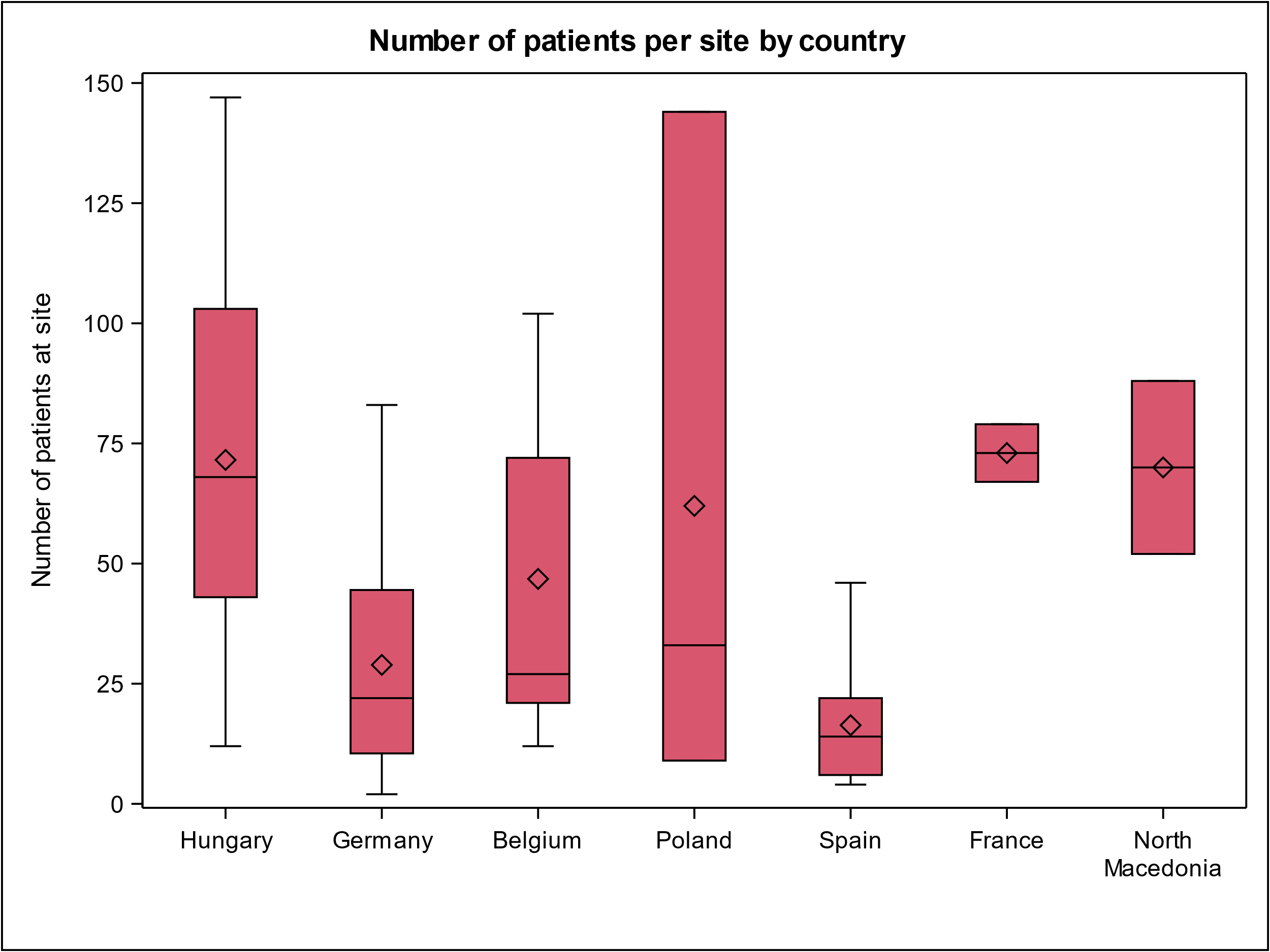
**

Figure shows the distribution of patient numbers per site by country for countries with at least 100 patients enrolled. The box indicates the interquartile range (IQR) (the range of values between the 25^th^ – 75^th^ percentiles); the line inside the box indicates the median; and the diamond indicates the mean. The whiskers indicate the range of values outside the IQR but within 1.5x IQR.

# Figure S3: Post-dilatation balloon pressure (categorized)

Figure shows the distribution of post-dilatation balloon pressure (categorized as No post-dilatation / ≤ 11 atm / > 11 atm) within country for countries with at least 100 patients enrolled. Atm = atmosphere.

# Figure S4: Odds ratio for MAE adjusted for number of patients per site (A) and adjusted for the post-dilatation balloon pressure (B)

Figure shows odds ratios (OR) with 95% confidence intervals (95% CI) for 30-day MAE following carotid artery stenting adjusted for (A) number of patients enrolled per site and (B) post-dilatation balloon pressure (categorized as No post-dilatation / ≤11atm / >11atm). Analysis includes countries with at least 100 enrolled subjects and Hungary serves as the reference country (OR = 1.0). P-value indicates overall significance of country-specific variation. MAE = Major Adverse Event (i.e. any death or stroke), atm = atmosphere.
